# From immunogenetic polymorphism to functional antitumor lymphocyte mitochondrial dynamics

**DOI:** 10.1186/2051-1426-3-S2-O13

**Published:** 2015-11-04

**Authors:** Roman Uzhachenko, Menaka Thounaojam, Anil Shanker

**Affiliations:** 1 Meharry Medical College, Nashville, TN, USA; 2 Vanderbilt-Ingram Cancer Center, Nashville, TN, USA

## 

The major histocompatibility complex and the leukocyte receptor complex are the two most polymorphic regions of the immune genome. Individuals with increasingly diverse repertoires of MHC class-I molecules have a greater potential for their natural killer (NK) cells to be more responsive. NK cell activity is under complex regulation of NK cell education that is fine-tuned by tumor microenvironment. NK cells with a greater number of inhibitory receptors that recognize the surrounding MHC class-I respond to stimuli better than NK cells with less recognition of the surrounding MHC. We investigated the mechanisms of local tumor antigen-specific T cell-NK cell collaboration, which appeared indispensable for the elimination of tumor cells, including antigen-deficient tumor escape variants that arise before metastasis. We observed in a mouse model of mastocytoma expressing a self-tumor-antigen P1A that effector CD8T cells provided a necessary “help” to dormant NK cells in eliciting their antitumor effector function. Bioluminescence imaging of mastocytoma tumors following adoptive transfer of P1A-specific T cells in RAG-/- and RAG-/-γc-/- mice showed that NK cell anti-tumor activity requires cytolytic T cells, whereas T cells can function independent of NK cells. In 2D and 3D co-culture systems, we observed that PMA/ionomycin-stimulated CD8T cells form multiple contacts with naïve NK lymphocytes. Data show that NK cells interacting with activated CD8T cells show an up-regulation of CD25 and CD69 expression mediated by intercellular contacts, and activation of NKG2D receptors and Stat2, Stat6, Jak1, Jak3, Tyk2, and PTEN signaling molecules with a decrease in the phosphorylation of Stat1, PKB/Akt, SAPK/JNK, p38. On the other hand, interacting NK cells down-regulate CD25 molecule expression on CD8T cells and promote differentiation of central-memory CD44+CD62L+T cells. CD8T cells display an elevation in the phosphorylation of Stat1 and down-regulation of Stat5 with stimulated PKB/Akt, Lck, mTOR, and p42/p44. Alterations in phosporylation status of multiple signaling proteins during CD8T–NK interaction suggest a cellular remodelling, whereby NK cells polarize activated CD8T cells towards a central-memory phenotype and activated CD8T lymphocytes induce naive NK cells towards effector/regulatory phenotype. Moreover, significant changes in the cytosolic and mitochondrial Ca2+, production of mitochondrial ROS, mitochondrial membrane potential, mitochondrial permeability transition pore, and synthesis of nitric oxide and non-protein thiols (mostly, reduced glutathion) were observed in a reciprocal CD8T–NK interaction. These findings highlight the importance of mitochondrial activity in the re-modeling of activation signaling and memory differentiation of interacting CD8T cells and NK cells, with a potential to refine cancer immunotherapeutic strategies.

